# Recurrent Adenosine-Sensitive Supraventricular Tachycardia Associated With the Menstrual Cycle in a Middle-Aged Woman: A Case Report of Typical Atrioventricular Nodal Reentrant Tachycardia (AVNRT)

**DOI:** 10.7759/cureus.103026

**Published:** 2026-02-05

**Authors:** Stanislaw Szymkiewicz

**Affiliations:** 1 Department of Urology, Janusz Korczak Provincial Specialist Hospital in Słupsk, Słupsk, POL

**Keywords:** adenosine, avnrt, catheter ablation, hormonal influence on arrhythmia, menstrual cycle, supraventricular tachycardia

## Abstract

Atrioventricular nodal reentrant tachycardia (AVNRT) is the most common cause of paroxysmal supraventricular tachycardia in adults and frequently presents with sudden-onset palpitations and regular narrow-complex tachycardia. Although generally considered benign, recurrent symptomatic episodes often lead to repeated emergency department visits and reduced quality of life, warranting definitive therapy. We report the case of a 42-year-old woman who presented with several hours of palpitations and was found to have a regular narrow-complex tachycardia at approximately 200 beats per minute without visible P waves. Subtle pseudo r’ deflections in lead V1 and pseudo S waves in the inferior leads were noted, consistent with typical slow-fast AVNRT. Vagal maneuvers were ineffective, and intravenous adenosine at a dose of 12 mg resulted in transient atrioventricular block followed by the prompt restoration of sinus rhythm. Post-conversion electrocardiography demonstrated normal atrioventricular conduction without evidence of ventricular preexcitation, and transthoracic echocardiography confirmed the absence of structural heart disease. The patient reported multiple similar episodes over the preceding months, frequently occurring around the onset of menstruation, suggesting a potential influence of hormonal fluctuations on arrhythmia susceptibility. Given the recurrent and symptomatic nature of the episodes, she was referred for electrophysiological study and catheter ablation, which is recommended as first-line definitive therapy for AVNRT. This case highlights the classic electrocardiographic features and adenosine sensitivity of AVNRT, supports early referral for curative ablation in recurrent cases, and draws attention to possible hormonal modulation as a trigger for supraventricular tachycardia in susceptible patients.

## Introduction

Atrioventricular nodal reentrant tachycardia (AVNRT) is the most frequent mechanism of paroxysmal supraventricular tachycardia, accounting for approximately 60% of cases in adults. It results from reentry within or adjacent to the atrioventricular node utilizing dual atrioventricular nodal pathways, typically a fast and a slow pathway, with different conduction velocities and refractory periods [1,2]. Clinically, AVNRT typically presents with sudden-onset palpitations, and electrocardiography often demonstrates a regular narrow-complex tachycardia with absent or retrograde P waves.

Acute termination is commonly achieved with vagal maneuvers or intravenous adenosine. In patients with recurrent or symptomatic episodes, catheter ablation of the slow pathway is highly effective and is recommended as first-line definitive therapy according to the current European Society of Cardiology guidelines, with long-term success rates exceeding 95% and low complication rates reported in large clinical series [1,3].

While AVNRT is not traditionally considered hormonally mediated, emerging evidence suggests that fluctuations in autonomic tone and sex hormone levels during the menstrual cycle may influence arrhythmia susceptibility in some women [4,5]. Several reports have described clustering of supraventricular arrhythmias during specific phases of the menstrual cycle; however, these observations remain largely descriptive, and mechanistic confirmation is lacking. We present a case of recurrent AVNRT temporally associated with the onset of menstruation in a patient with a structurally normal heart.

## Case presentation

A 42-year-old woman presented to the emergency department with a several-hour history of palpitations that began suddenly at rest. She denied chest pain, dyspnea, syncope, or presyncope. She reported three similar emergency department presentations over the preceding three months, with additional self-limited episodes. The episodes most commonly occurred within the first 1-2 days of menstruation.

Her medical history included treated hypothyroidism and a remote ischemic stroke in 2022. There was no known structural heart disease. On examination, she was hemodynamically stable, and cardiovascular and pulmonary examinations were unremarkable.

**Figure 1:**
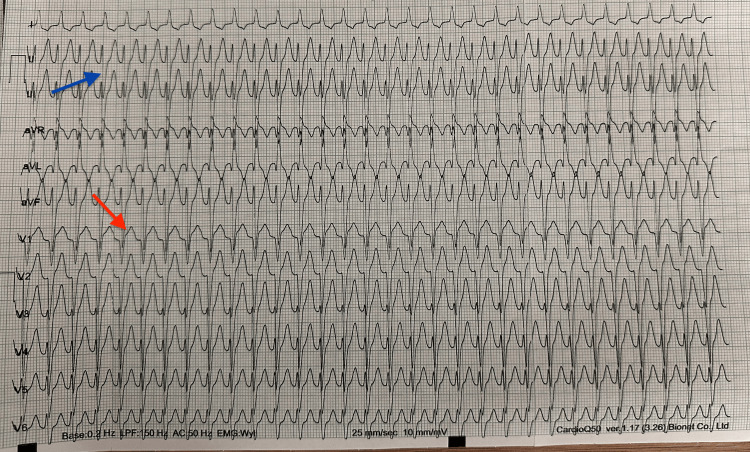
Electrocardiogram during supraventricular tachycardia at 200 beats per minute Twelve-lead electrocardiogram demonstrating a regular narrow-complex tachycardia at approximately 200 beats per minute. P waves are not clearly visible, and subtle pseudo r′ deflections in lead V1 (indicated by the red arrow) as well as pseudo S waves in the inferior leads (indicated by the blue arrow) can be appreciated, consistent with typical slow-fast AVNRT. AVNRT: atrioventricular nodal reentrant tachycardia

Electrocardiography demonstrated a regular narrow-complex tachycardia at approximately 200 beats per minute without visible P waves. Subtle pseudo r′ deflections in lead V1 and pseudo S waves in the inferior leads were noted, suggestive of AVNRT (Figure 1). According to recommended management algorithms for stable narrow-complex supraventricular tachycardia, vagal maneuvers are considered first-line therapy, followed by intravenous adenosine if non-pharmacologic measures fail [1,2]. Unilateral carotid sinus massage was performed under continuous electrocardiographic and hemodynamic monitoring without the termination of the arrhythmia.

In our institution, the initial dose of adenosine for stable supraventricular tachycardia is routinely 12 mg, as lower doses are frequently ineffective in adult patients; this practice reflects local emergency department protocols despite guideline recommendations to start with 6 mg, and no patient-specific factors necessitating dose escalation were identified. Intravenous adenosine 12 mg was administered, resulting in transient atrioventricular block followed by the restoration of sinus rhythm (Video 1). The transient atrioventricular block and abrupt termination of tachycardia further supported an atrioventricular node-dependent reentrant mechanism [2].

**Video 1:** Termination of supraventricular tachycardia after intravenous adenosine administration Video recording of a 12-lead electrocardiogram demonstrating regular narrow-complex supraventricular tachycardia followed by transient atrioventricular block after the administration of 12 mg intravenous adenosine and subsequent restoration of sinus rhythm. The abrupt termination of tachycardia confirms an atrioventricular node-dependent reentrant mechanism, consistent with typical AVNRT. AVNRT: atrioventricular nodal reentrant tachycardia

**Figure 2:**
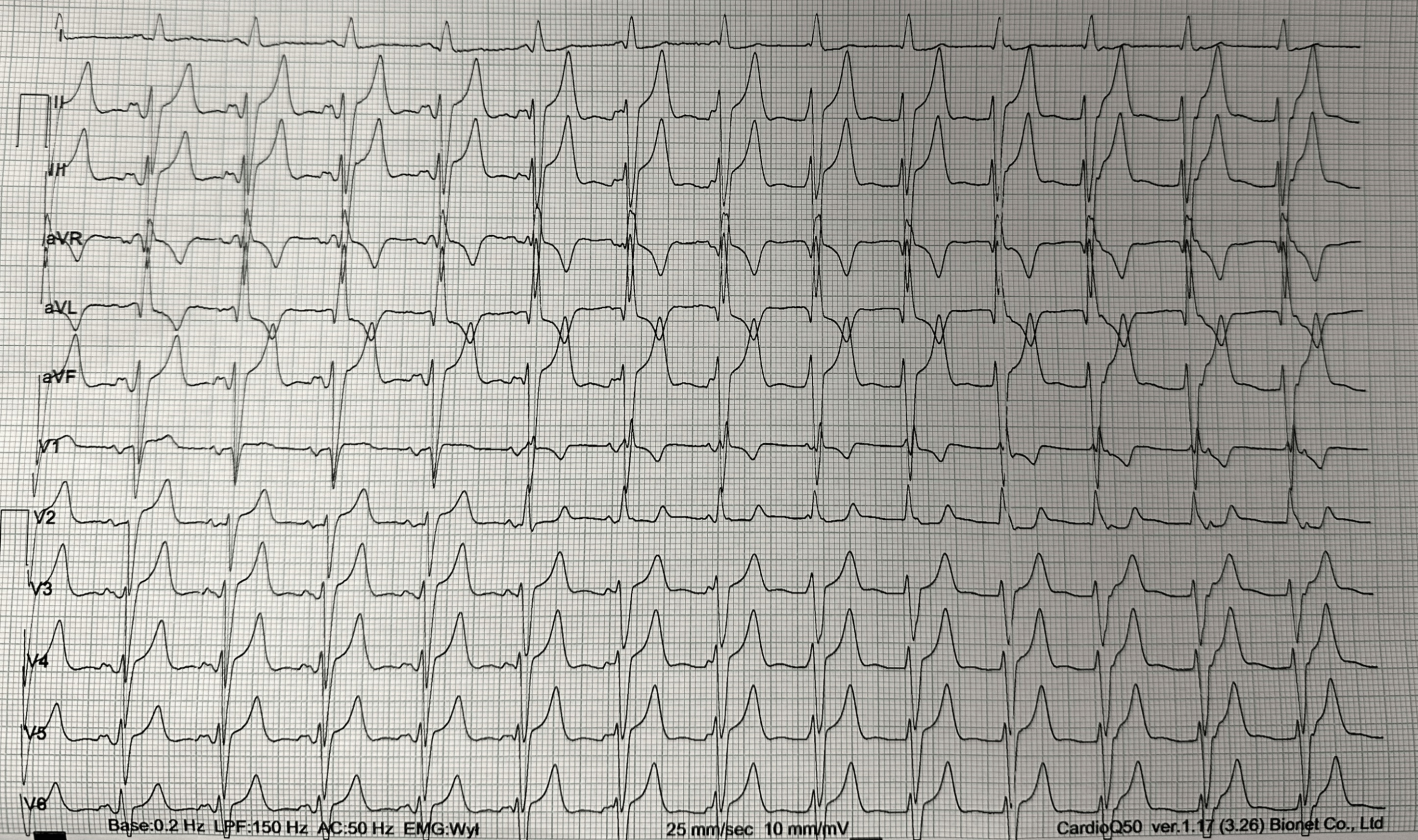
Electrocardiogram after conversion to sinus rhythm following adenosine administration Twelve-lead electrocardiogram obtained after intravenous adenosine showing the restoration of sinus rhythm with normal atrioventricular conduction, narrow QRS complexes, and no evidence of ventricular preexcitation. These findings further support an atrioventricular node-dependent mechanism of the preceding tachycardia.

Post-conversion electrocardiography showed sinus rhythm with normal PR interval, narrow QRS complexes, and no evidence of ventricular preexcitation (Figure 2). Laboratory studies, including electrolytes and inflammatory markers, were within normal limits. Thyroid function tests are not routinely available in the emergency department at our institution and are typically reserved for cases with clinical suspicion of thyroid storm; therefore, thyroid-stimulating hormone (TSH), free T3, and free T4 were not obtained during the current visit, although thyroid dysfunction was considered as a potential contributing factor. However, outpatient thyroid function testing had been recommended after a previous emergency visit one month earlier, and according to the patient's report, subsequent ambulatory evaluation demonstrated thyroid hormone levels within the reference range.

Transthoracic echocardiography demonstrated preserved left ventricular systolic function (ejection fraction 60%), no regional wall motion abnormalities, normal right ventricular function (tricuspid annular plane systolic excursion (TAPSE) 25 mm), and no significant valvular disease or pericardial effusion. Given recurrent symptomatic episodes, the patient was referred to an arrhythmia clinic for electrophysiological study and the consideration of catheter ablation, consistent with guideline recommendations for patients with recurrent AVNRT [1,3].

## Discussion

AVNRT is the most common form of paroxysmal supraventricular tachycardia, accounting for approximately 60% of cases and occurring more frequently in women, often presenting in young or middle adulthood [1,3]. The characteristic electrocardiographic features include a narrow QRS complex tachycardia with absent or pseudo r′ waves in lead V1 and pseudo S waves in the inferior leads, reflecting simultaneous atrial and ventricular activation [1].

Adenosine remains the first-line pharmacologic therapy for the acute termination of atrioventricular node-dependent supraventricular tachycardias and has demonstrated high efficacy and a favorable safety profile in multiple clinical studies, including emergency department-based trials and meta-analyses [2,6,7]. In hemodynamically stable patients, vagal maneuvers followed by adenosine are recommended as first-line therapies for the acute termination of supraventricular tachycardia, with synchronized cardioversion reserved for unstable cases [2,8].

Abrupt termination of tachycardia after transient atrioventricular block is characteristic of atrioventricular node-dependent reentrant mechanisms and helps distinguish AVNRT from focal atrial tachycardia, highlighting the diagnostic utility of adenosine in supraventricular tachycardia [2,9]. Orthodromic atrioventricular reentrant tachycardia was considered less likely given the absence of ventricular preexcitation on baseline electrocardiography. In the present case, adenosine administration resulted in the immediate termination of the arrhythmia and restoration of sinus rhythm, supporting the diagnosis of AVNRT.

Catheter ablation of the slow pathway offers long-term success rates exceeding 95% with a low risk of complications and is considered the definitive treatment for patients with recurrent or symptomatic AVNRT [1,3]. Similar high success rates and low complication profiles of slow pathway ablation in AVNRT have also been reported in observational studies and case-based analyses [10]. Current guidelines recommend catheter ablation as a first-line definitive therapy in patients with frequent episodes, poor tolerance of arrhythmia, or a preference for non-pharmacologic management [3]. The patient in this case was therefore referred for electrophysiological evaluation and potential ablation following stabilization.

The association between hormonal fluctuations and supraventricular tachyarrhythmias has been previously described, with some studies suggesting increased susceptibility during the luteal phase of the menstrual cycle, potentially due to progesterone-mediated autonomic and electrophysiologic effects [4,5]. Although causality cannot be established in a single case, the temporal association between symptom exacerbation and the menstrual cycle in this patient may support this observation; however, this finding remains observational and hypothesis-generating, and prospective or mechanistic studies are needed to further clarify this relationship.

This case highlights the importance of recognizing the typical electrocardiographic features of AVNRT and the dual diagnostic and therapeutic role of adenosine in the emergency department. Prompt identification and appropriate management can lead to rapid symptom resolution and facilitate timely referral for definitive treatment.

## Conclusions

This case illustrates the typical presentation and electrocardiographic features of AVNRT and highlights the effectiveness of adenosine in both diagnosis and acute management. In patients with recurrent symptomatic episodes, referral for electrophysiological study and catheter ablation is warranted as definitive therapy. Hormonal influences related to the menstrual cycle may represent an underrecognized and observationally suggested trigger for supraventricular tachycardia; however, this association remains hypothesis-generating and warrants further prospective and mechanistic investigation, particularly in women with recurrent, cyclically patterned arrhythmia episodes.

## References

[1] Brugada J, Katritsis DG, Arbelo E (2020). 2019 ESC guidelines for the management of patients with supraventricular tachycardia: the Task Force for the management of patients with supraventricular tachycardia of the European Society of Cardiology (ESC). Eur Heart J.

[2] Bibas L, Levi M, Essebag V (2016). Diagnosis and management of supraventricular tachycardias. CMAJ.

[3] Yaminisharif A, Davoodi G, Kasemisaeid A, Farahani AV, Ghazanchai F, Moghaddam M (2010). Radiofrequency catheter ablation of atrioventricular nodal reentrant tachycardia: success rates and complications during 14 years of experience. J Tehran Heart Cent.

[4] Schreuder MM, Sunamura M, Roeters van Lennep JE (2019). Supraventricular tachycardia and the menstrual cycle. Case Rep Womens Health.

[5] Dogan M (2020). Electrophysiological changes during menstrual cycle. Sex and Cardiac Electrophysiology.

[6] Feng X, Liu J (2025). Efficacy and safety of adenosine for supraventricular tachycardia: a meta-analysis utilizing BioMedGPT-LM-7B. BMC Cardiovasc Disord.

[7] Marco CA, Cardinale JF (1994). Adenosine for the treatment of supraventricular tachycardia in the ED. Am J Emerg Med.

[8] Patti L, Horenstein MS, Ashurst JV (2025). Supraventricular tachycardia. StatPearls.

[9] Ector J, Haemers P, Garweg C, Willems R (2020). Diagnosis and treatment of atrioventricular nodal reentrant tachycardia: a case report illustrating clinical management and ablation strategy. Eur Heart J Case Rep.

[10] Gupta A, Lokhandwala Y, Rai N, Malviya A (2021). Adenosine-a drug with myriad utility in the diagnosis and treatment of arrhythmias. J Arrhythm.

